# Identification of two ISG15 homologues involved in host immune response against RGNNV in Asian seabass (*Lates calcarifer*)

**DOI:** 10.1016/j.fsirep.2022.100054

**Published:** 2022-03-08

**Authors:** R.S. Krishna Priya, Avinash Premraj, K.C. Sivakumar, T.P. Sajeevan

**Affiliations:** a National Centre for Aquatic Animal Health, Cochin University of Science and Technology, Fine Arts Avenue, Kochi, 682 016, Kerala, India; b Camel Biotechnology Centre, Presidential Camels and Camel Racing Affairs Centre, Department of the President's Affairs, PO Box 17292, Al Ain, United Arab Emirates; c Rajiv Gandhi Centre for Biotechnology, Poojappura, Thiruvananthapuram, Kerala 695 014 India

**Keywords:** *Lates calcarifer*, Interferon-stimulated gene, RGNNV, Antiviral, Teleost

## Abstract

Interferon Stimulated Gene (ISG)15 is a ubiquitin-like protein that is induced upon viral infections. Our study reports the identification of two homologues of ISG15 in the Asian seabass designated LcISG15A and LcISG15B. The cloned LcISG15A cDNA fragment contained a 474 bp ORF encoding a 157 amino acid protein whereas LcISG15B featured a 498 bp ORF encoding a slightly longer protein of 165 amino acids. Both proteins featured the two tandem ubiquitin-like domains and the C-terminal LRGG motif characteristic of ISG15. The LcISG15B protein has a 10-amino acid C-terminal extension after the LRGG motif. Molecular docking studies revealed that LcISG15A showed more conformational variability of the ubiquitin domains and catalytic function than LcISG15B. The *Lates* ISG15A and ISG15B genes, reside close in the genome, share the same basic structure with two exons and an intron, but only the second exon encoding the protein. These genes also featured the IFN-stimulatory response elements (ISRE) in the promoter region and ATTTA instability motif in the 3′ UTR region. Leukocyte-rich organs such as the head kidney, heart, spleen, and gill showed higher levels of ISG15A and ISG15B basal expression. Poly (I:C) injection rapidly upregulated the transcription of both the ISG15 genes in these tissues in *Lates*. In-vivo viral infection by red-spotted grouper nervous necrosis virus also induced upregulation of ISG15 genes in the head kidney, spleen, heart and gill. These findings indicate that the two ISG15 homologues may play a crucial role in innate antiviral immunity and could be used to improve prophylactic strategies and develop species-specific immunological tools for *Lates calcarifer*.

## 1 Introduction

Type I IFNs are involved in the first line of defense against viral infection in vertebrates. The secretion of type I IFN produces an antiviral state by transcription of numerous interferon-stimulated genes (ISGs), including ISG15 through JAK/STAT pathway [1,2]. Interferon stimulated gene (ISG)15 is a ubiquitin-like protein with moonlighting actions that is highly up-regulated during viral infections [3,4]. In mammals, ISG15 contains two tandem ubiquitin-like domains (UBL) and a conserved C-terminal LRGG sequence required for covalent conjugation of ISG15 proteins with host and viral proteins in a process called ISGylation [5]. ISGylation does not target proteins for proteasome degradation like ubiquitination [6]. However, it activates or suppresses the function of target proteins [7,8] by a series of enzymatic reactions that lead to enzymatic degradation and subcellular localisation of target proteins in mammals [9,10]. Apart from its role as an intracellular protein modifier, ISG15 also exists in a secreted form and has been reported to induce IFN-γ production in natural killer (NK) cells and T lymphocytes [11]. It is believed that ISG15 appeared in gnathostomes and diverged, but interestingly it is absent in birds and the gene has not been reported from reptiles or amphibians [12,13]. Recently, reports have emerged of genome-predicted ISG15 genes in some reptiles and detection of ISG15 transcripts in amphibians such as Chinese giant salamanders [13], [14], [15].

ISG15 has been reported in many fish species, including Japanese flounder, black rockfish, Atlantic salmon, Atlantic cod, Channel catfish, crucian carp, turbot, sea bream and seabass [16]. As in other vertebrates, most fish have a single copy of the *ISG15* gene, while a few possess more than one [17].

The Asian seabass (*Lates calcarifer)* is a commercially important marine or brackish water fish species widely cultured in India. Viral diseases are the most important threat associated with the aquaculture of Asian seabass. Among the viral pathogens of *Lates*, the nervous necrosis virus (NNV) cause mass mortality of the larvae and often hamper the hatchery operations [18]. Nervous Necrosis Virus (VNN) or betanodavirus infection causes viral encephalopathy and retinopathy (VER) in fishes. Betanodavirus genome has two linear positive-sense single-stranded RNA. Structurally these zoonotic viruses are non-enveloped with icosahedral shape [19,20]. Infected fishes have visible symptoms of dark colour and abnormal swimming behaviour. They show intensive vacuolation of the retina and nervous system, causing severe neurological disorders [21]. This disease was reported to cause about 100 % mortality in larvae and juvenile of affected fish species leading to severe economic losses [22]. The nodavirus infections were reported to affect about 120 species, including the Asian seabass [21,23].

Considerable research has been carried out in teleost fishes like zebrafish on ISGs that are considered key effector molecules that limit viral replication by acting at different points and preventing disease progression. Upon betanodavirus infection, ISG15 is one of the earliest and most abundantly expressed Interferon-stimulated genes (ISGs) in fishes. A correlation between virus levels and ISG15 mRNA expression has been found in the betanodavirus-infected brain and kidney of *Dicentrarchus* [24,25]*.* Nodavirus infection poses a substantial threat to aquaculture in Australia and Southeast Asia including India. Species-specific variations in the protein sequence of ISG15 have been reported to affect the interaction between the virus and the host, as well as the immune evasion by many pathogenic viruses [3]. Identifying species-specific immune genes such as ISG15 in seabass can shed light on the host-viral protein interactions during viral infection and help develop better antiviral strategies.

We examined the gene structure and expression to identify the function of the *ISG15* gene homologues in *Lates* innate antiviral immunity. To understand the role of LcISG15 homologues in innate immune response, we challenged Asian seabass *Lates calcarifer* with poly (I:C) and Red-spotted Grouper Nervous Necrosis Virus (RGNNV), which causes massive mortality in aquaculture. This would pave the way in developing strategies to control viral diseases in this commercially important species.

## 2 Materials and methods

### 2.1 Ethics statement

All animal experimental procedures were approved by the Institutional Animal Ethics Committee of Cochin University of Science and Technology (CUSAT), Kerala, India. The animals used in the experiments were all cultured stock, anesthetised with clove oil, and euthanised during the experiment.

### 2.2 Fishes, cell-line and virus

The Asian seabass (approximately 50 g body weight) were purchased from Rajiv Gandhi Centre for Aquaculture (RGCA) complex at Vallarpadam, Kochi, India and was acclimatised for two weeks in a laboratory aquaculture facility. They were maintained in tanks at 15 ppt salinity and temperature 28-30°C. During the period of acclimatisation, the fishes were closely monitored for any visible clinical symptoms of the disease (like darkening of skin, abnormal swimming behaviour, etc). Before the experiment, 2-3 fishes were randomly selected from each tank dissected out, organs were pooled for PCR-based detection of the RGNNV virus. After confirming that the tested samples were RGNNV negative in PCR, we proceeded for further experiments. After acclimatisation, the healthy fishes were selected and euthanised after anesthetising with clove oil. For tissue distribution studies, nine fishes were dissected out, and tissues (head kidney, spleen, gill, heart, muscle, blood, fin and brain) were pooled and stored in RNAlater (Sigma).

SISK (Sahul Indian Seabass Kidney) cell-line was maintained at 28°C in L-15 (Leibovitz) medium (HiMedia, India) supplemented with 15% Fetal Bovine Serum (FBS, HiMedia)[26]. RGNNV previously isolated from an infected seabass was propagated in SISK cell line using L-15 media supplemented with 2% FBS. When extensive cytopathic effects were evident, the cells were freeze-thawed thrice to release the virus, the lysate was collected and centrifuged at 5000 rpm. The collected supernatant containing the virus was stored at -80°C and was used for experimental infection. For virus infection experiments the viral titers were measured by a TCID_50_ (50% tissue culture infective dose) assay according to the method of Reed and Muench [27].

### 2.3 Challenge experiments with poly(I:C) and RGNNV virus

For analysis of gene expression following poly(I:C) injection and RGNNV infection, the fishes approximately 50 grams of weight were divided into three groups of thirty numbers each, anaesthetised and intraperitoneally injected with 100µl of poly (I:C) (Sigma P1530) diluted in PBS at a dose of 100µg per 100g body weight of fish or 100 µl of RGNNV at a concentration of 1 × 10^6^ TCID_50_/ml. Tissues from six animals from each group were pooled at 6,12,24,48 and 72 hours of post-injection and were analysed for each time point. The fishes in the control group were injected with an equal amount of PBS. The head kidney, spleen, heart and gill samples were collected at 6,12,24,48 and 72 hours of post-injection for gene expression analysis from both the challenge experiments.

### 2.4 Molecular cloning of ISG15 homologues

Total RNA was extracted from the tissue samples using TRIzol Reagent (Invitrogen, Waltham, MA, USA), treated with DNase to remove genomic DNA contamination, quantified using Nanodrop (Implen, GmbH, München, Germany). Two micrograms of RNA were reverse transcribed using M-MuLV reverse transcriptase (NEB, Ipswich, MA, USA), primed with oligo(dT) primer. Primers for amplifying ISG15 homologues were designed based on a partially annotated whole-genome sequence of *Lates* available from NCBI (Table 1). *Lates* gill cDNA was amplified with these primers using Emerald master mix (TaKaRa, Japan). Genomic DNA was isolated from gill by DNA-Xpress reagent (HiMedia Labs, Mumbai, India) according to the manufacturer's protocol. The isolated DNA was used to amplify the ISG15 gene of *Lates* by using designed primers (Table 1). The amplification conditions were: 94˚C for 2 minutes followed by 35 cycles of 94˚C for 30 seconds, 58˚C for 30 seconds, extension at 72˚C for 1 minute followed by a final extension at 72˚C for 7 minutes. The amplified products were cloned into pGEM-T Easy Vector (Promega, Madison, WI USA) and recombinant plasmids were sequenced using BigDye Terminator Cycle sequencing (Thermo Fisher, Waltham, MA, USA) in an ABI 3730 DNA Analyzer (Applied Biosystems, Foster City, CA, USA). We compared the cloned genomic *Lates ISG15A* and *ISG15B* sequences to their corresponding cDNA sequences using the Splign cDNA-to-genomic alignment tool (NCBI) to determine exon-intron organisation.

**Table 1.** Oligonucleotide PCR primers designed for the amplification of LcISG15 homologues and quantitative real-time PCR.

**Name**	**Sequence (5’- 3’)**	**Application**
LcISG15AF	CACAGCTGCTGCAGAGCATCGTC	Cloning
LcISG15AR	GTCCTCAGCCTCCTCTCAGACG	Cloning
LcISG15BF	CGAGTCAACCTTCAAGCGCAGC	Cloning
LcISG15BR	GATCAGAGTGCTTTGTGTTCACG	Cloning
LcISG15AqF	TGCAGGTGAGCCAGCAGAGGC	Real time PCR
LcISG15AqR	CACAAGTCGATGGTGCTCAGCG	Real time PCR
LcISG15BqF	GTGCCGACGGACCAGTTCTATC	Real time PCR
LcISG15BqR	CACATGTTGATGGTGCTCATCTC	Real time PCR
Lc βactin qF	AGCATCATGAAGTGCGACGTC	Real time PCR
Lc βactin qR	CTCCTTCTGCATCCTGTCAGC	Real time PCR

### 2.5 Sequence analysis

The putative open reading frame (ORF) and predicted amino acid sequence of the cloned *Lates* ISG15 were identified using ORF finder (https://www.ncbi.nlm.nih.gov/orffinder/). The nucleotide sequences and the predicted amino acid sequences of the cloned *Lates* ISG15 were aligned with the corresponding sequences of other fishes obtained from the NCBI GenBank using the CLUSTAL W program. The ScanProsite (https://prosite.expasy.org/scanprosite/) was used to predict the domains in the derived protein sequences.

The cloned *ISG15A* and *ISG15B* genomic sequences were aligned against the *Lates* genome. The cloned sequences were found to be contained within the *Lates calcarifer* isolate ASB-BC8 unitig_5945_quiver, whole genome shotgun sequence NCBI Acc No LLXD01000096.1. The sequences flanking the cloned *Lates ISG15* gene sequences were identified and scanned for regulatory elements. The 3′ UTR was also deduced from the genomic alignment after identifying the Poly A signal sequence motif in the genomic sequence.

### 2.6 Phylogenetic analysis and secondary structure prediction

Phylogenetic analysis was carried out using the Maximum Likelihood method, and phylogenetic trees were constructed using the neighbour-joining method with MEGA 11 program [28] with 500 bootstrap replications to create the phylogenetic trees based on the protein distance in the aligned *Lates* and other ISG15 amino-acid sequences. The evolutionary distances were calculated using the Dayhoff model. The secondary structure was predicted by PSIPHRED (http://bioinf.cs.ucl.ac.uk/psiphred/), and the SWISS-MODEL protein homology-modelling server (https://swissmodel.expasy.org/) was used to predict the 3-D homology model of *Lates* ISG15A and ISG15B proteins was using the crystal structure of ubiquitin-like protein ISG15 (PDB:6bi8).

### 2.7 Normal Mode Analysis (NMA) of LcISG15A and LcISG15B

The NMA package written in R language using the Bio3D [29] was used for performing all-atom elastic network model *normal modes* calculation of the predicted models of LcISG15A and LcISG15B. Structural models of LcISG15A and LcISG15B obtained through homology modelling served to produce NMA ensembles. The ensembles produced by the NMA algorithm include eigenvectors and mode fluctuations for the isoforms that can be easily analysed and compared. The NMA ensemble can be performed on the aligned structures with the nma () function. This function contains a deformation.nma () object that represents the aligned eigenvectors, the mode fluctuations, and all pair-wise root mean square values for each component. These results facilitate a direct comparison of the elasticity patterns of these proteins.

### 2.8 Analysis of *ISG15A* and *ISG15B* mRNA expression in different tissues in healthy seabass

Quantitative real-time PCR analysis of *LcISG15A and LcISG15B* mRNA expression in *Lates* tissues were performed in Step OnePlus Real-Time PCR machine (Applied Biosystem) using SYBR Premix Ex Taq (Takara Bio, Shiga, Japan) with designed primers (Table 1). For tissue distribution studies of *Lates* mRNA, total RNA was isolated from tissues (head kidney, heart, muscle, spleen, blood, gill, fin, and brain), and cDNA was synthesised. The qRT PCR mix consists of 5 µl SYBR green master mix (TAKARA), 0.5 µl ROX dye, 2 µl of template cDNA, 0.4 µl of both forward and reverse primers and 1.7 µl of nuclease-free water. The PCR conditions for LcISG15A was:1 cycle of 5 min at 95°C, followed by 39 cycles of 94°C for 15 seconds, 58°C for 40 seconds and LcISG15B was: 1 cycle of 5 min at 95°C, followed by 39 cycles of 94°C for 30 seconds, 60°C for 60 seconds. The gene expression was normalised using *Lates* β-actin as an internal reference and calibrated to the brain by using the 2^−ΔΔCt^ method.

### 2.9 Quantitative real-time PCR analysis of poly(I:C) and RGNNV challenged tissues

The total RNA was isolated from poly(I:C) treated and RGNNV infected tissues at 6,12,24,48, and 72 hours for the expression studies. About two micrograms of total RNA was used to synthesise cDNA. Gene expression was normalised using *Lates* β-actin as an internal reference using 2^−ΔΔCT^ and expressed as fold change by comparing the normalised gene expression level of treated groups with that of control. All data were represented as relative mRNA expression (mean ± SEM).

### 2.10 Statistical analysis

Statistical analysis was carried out using SPSS 20 software. Differences in the data were compared by using one-way ANOVA followed by Tukey's post-hoc test for multiple comparisons. Differences were considered significant at p < 0.05.

## 3 Results

### 3.1 Cloning of two distinct *Lates calcarifer* ISG15 cDNA

The protein-coding region of *Lates calcarifer* ISG15A and ISG15B was amplified using designed primers from gill cDNA. The cloned LcSIG15A processes 592 bp fragment contained the ISG15 ORF (474 bp) encoding for a 157 amino acid (Fig. 1a). LcISG15B cDNA contained a 651 bp DNA fragment with a longer open reading frame of 498 bp encoding for a 165 amino acid protein (Fig. 1b). The cDNAs sequences encoding the *Lates* ISG15A and ISG15B were deposited in the NCBI GenBank with accession numbers MH489512 and MH489513, respectively.

**Fig. 1a:**
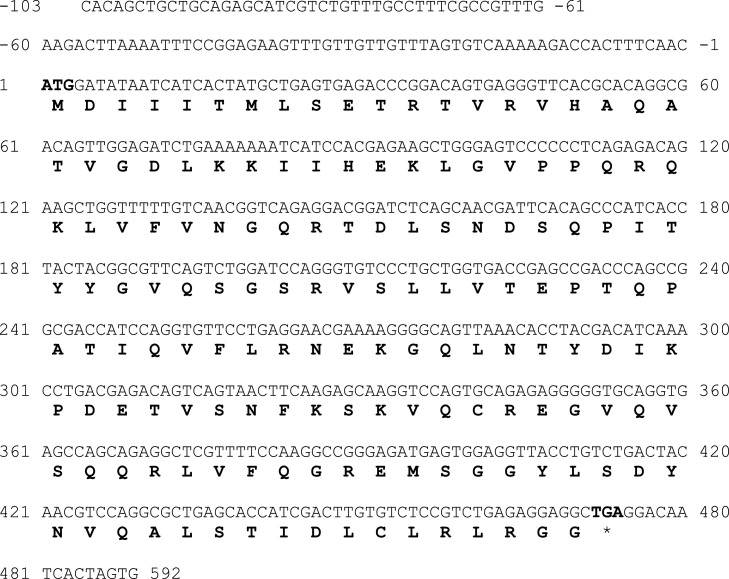
**Nucleotide sequence of the cloned 592 bp ISG15A fragment from the Asian seabass *Lates calcarifer*.** The 474 bp ISG15A protein-coding sequence (CDS) in the cloned cDNA fragment is marked with the start and stop codons highlighted. One-letter amino acid sequences are given in bold below the nucleotide sequences. First residue of the start codon is assigned number 1 and sequences upstream of ATG are given negative numbers. Single-letter amino acid translations are provided below the nucleotide sequence and the stop codon is denoted by an asterisk (*).

**Fig. 1b:**
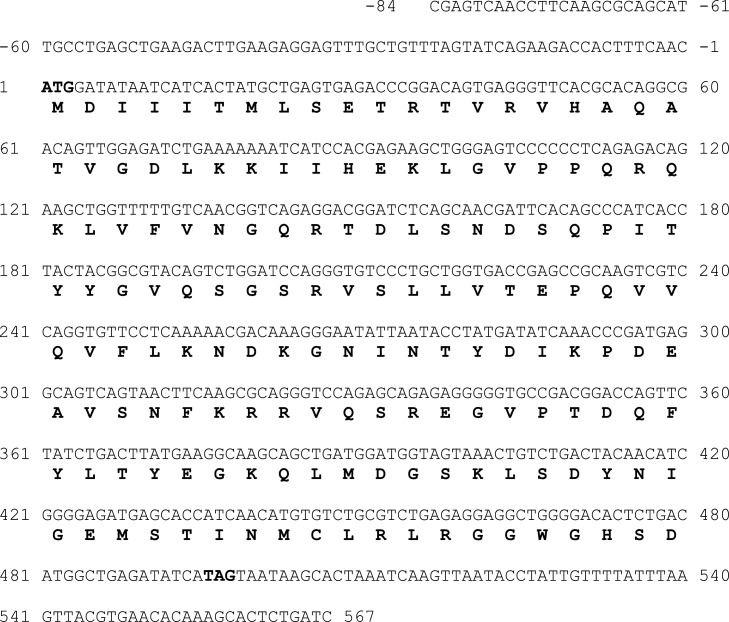
**Nucleotide sequence of the cloned 651 bp ISG15B cDNA fragment from the Asian seabass *Lates calcarifer*.** The ISG15B coding sequence (CDS) in the cloned cDNA fragment is marked with the start and stop codons highlighted. One letter amino acid sequences are given in bold below the nucleotide sequences. First residue of the Start codon is assigned number 1 and sequences upstream of ATG are given negative numbers. Single letter amino acid translations are provided below the nucleotide sequence and the stop codon is denoted by an asterisk (*).

Both the LcISG15A and LcISG15B proteins shared 73.8% sequence identity and 81.5% sequence similarity at the amino level. The eukaryotic signal peptide was not detected in both the predicted *Lates* ISG15 protein sequences. LcISG15A has a predicted molecular mass of 17.6 kDa and a calculated pI of 9.2, whereas LcISG15B has an estimated molecular mass of 18.6 kDa and a lower pI of 7.8. Both LcISG15 proteins featured two tandem Ubiquitin-like (UBL) domains as found in other ISG15s. The first domain was predicted to extend from 1-75 amino acids and is identical in both the LcISG15 proteins. The second Ubiquitin-like (UBL) domain has only 62% amino acid identity with 29 amino acid changes between LcISG15A and LcISG15B proteins. A cysteine residue is present in the linker between the two Ubiquitin-like (UBL) domains in most mammalian ISG15 sequences. However, it is absent in all fish ISG15 sequences, including *Lates*, whereas a proline residue is conserved at its place (Fig. 2a).

**Fig. 2a:**
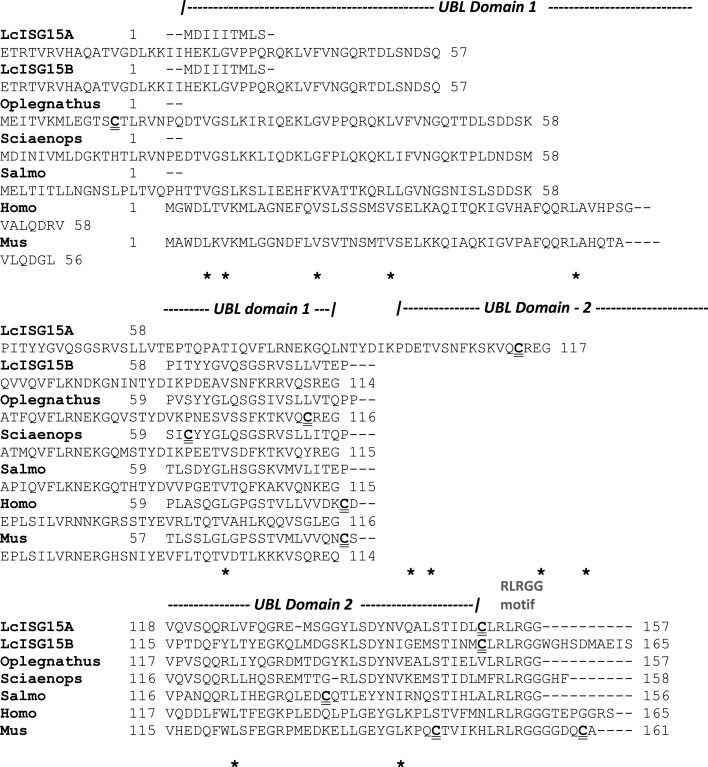
**Alignment of the deduced *Lates calcarifer* ISG15A and ISG15B amino-acid sequence with that of other fish, human and mouse homologs**. Residues strictly identical in all the species in the alignment are shown in black highlight whereas similar residues are marked in grey highlight. The two Ubiquitin-like domain (UBL) predicted in the sequence is marked. The six invariant aliphatic residues found in each of the UBLdomains are marked below by an asterisk (*). The RLRGG motif at the C-terminus is also marked. The cysteine residues are marked in bold and with a double underline.

Both the ISG15 proteins showed the conservation of the characteristic LRLRGG motif at the C-terminal found in other ISG15. Interestingly, the LcISG15B variant has ten additional amino acids after the LRLRGG motif at the C-terminus of the protein (Fig. 2a).

Compared to other reported fish ISG15s, the LcISG15A protein had the maximum amino acid similarity with ISG15 homologues from two perciform fishes - *Oplegnathus fasciatus* (86%) *and Sciaenops ocellatus* (82%). The corresponding similarity values for LcISG15B were 78% and 77%. With other fishes like *Sebastes schlegelii* (Scorpaeniformes); *Scophthalmus maximus* and *Paralichthys olivaceus* (Pleuronectiformes), the LcISG15A and LcISG15B have amino acid similarity ranging from 79 to 75 %. However, with the human ISG15, the amino acid similarity was only 30%.

LcISG15A and LcISG15B homologues share conformational similarities with two ubiquitin-like domains (I and II) at both termini, as observed in the crystal structure of the ubiquitin-like ISG15 protein (PDB:6bi8). Both comprise a central coil linker region that connects identical domains, each with a β-grasp fold of four β-sheets and one α-helix. The model predicts the presence of two cysteine ​​residues (Cys114 and Cys151) in the domain II of LcISG15A, which is expected to form disulfide bonds by consensus, and a single cysteine ​​(Cys149) is present in the domain II in LcISG15B. Cys151 and Cys149 residues are followed by the LRLRGG motif designate a distant activity and are dependent upon other nearby motifs or sequences in LcISG15A and ISG15B, respectively. Interestingly, no cysteine residues were detected in ubiquitin-like domain I in LcISG15A and LcISG15B. Therefore, both ubiquitin domains of ISG15 play several critical roles in ISGylation, which is unique between the ubiquitin and ubiquitin modifiers. Three-dimensional models of LcISG15A and LcISG15B proteins are represented in Fig. 2b.

**Fig. 2b:**
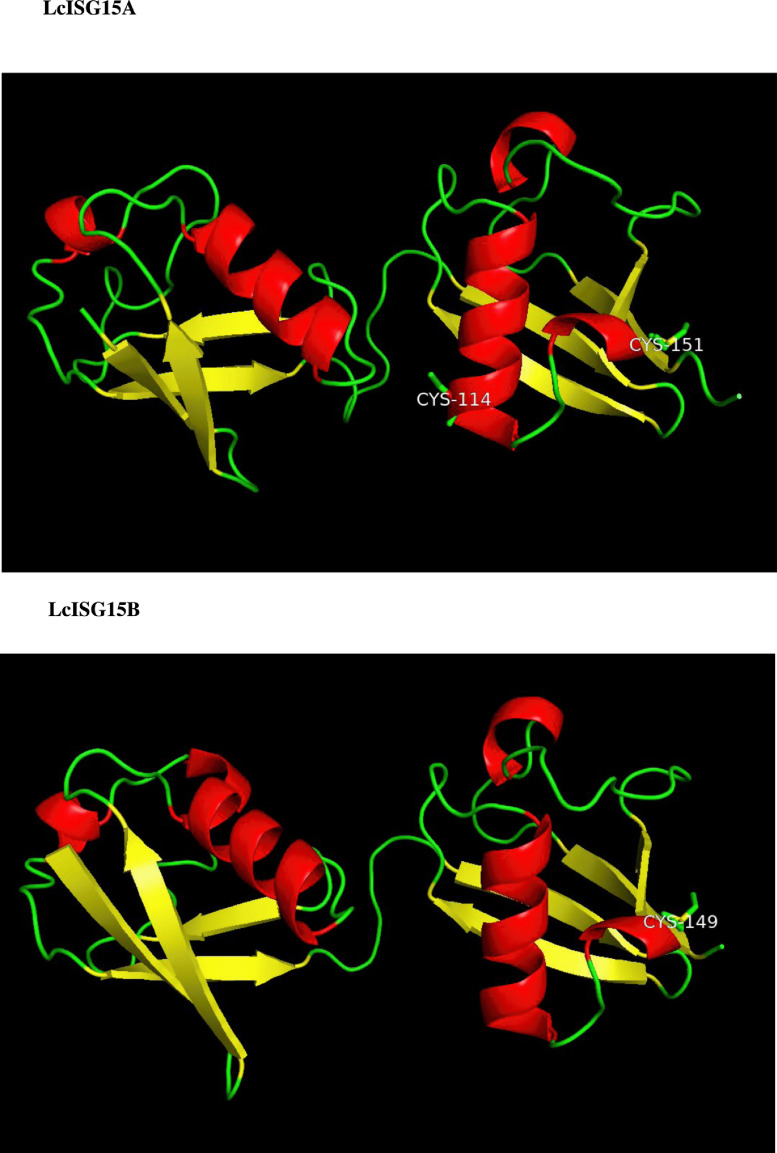
**Homology 3D model of the *Lates calcarifer* ISG15A & ISG15B proteins.** Homology model was generated using the SWISSMODEL protein homology-modeling server. Coloring is based on the secondary structure with helical regions in red, beta-strands in yellow and extended loop in green. The cysteine residues in the two *Lates* ISG15 proteins are marked.

### 3.2 Phylogenetic analysis of *Lates* ISG15

**Fig. 3:**
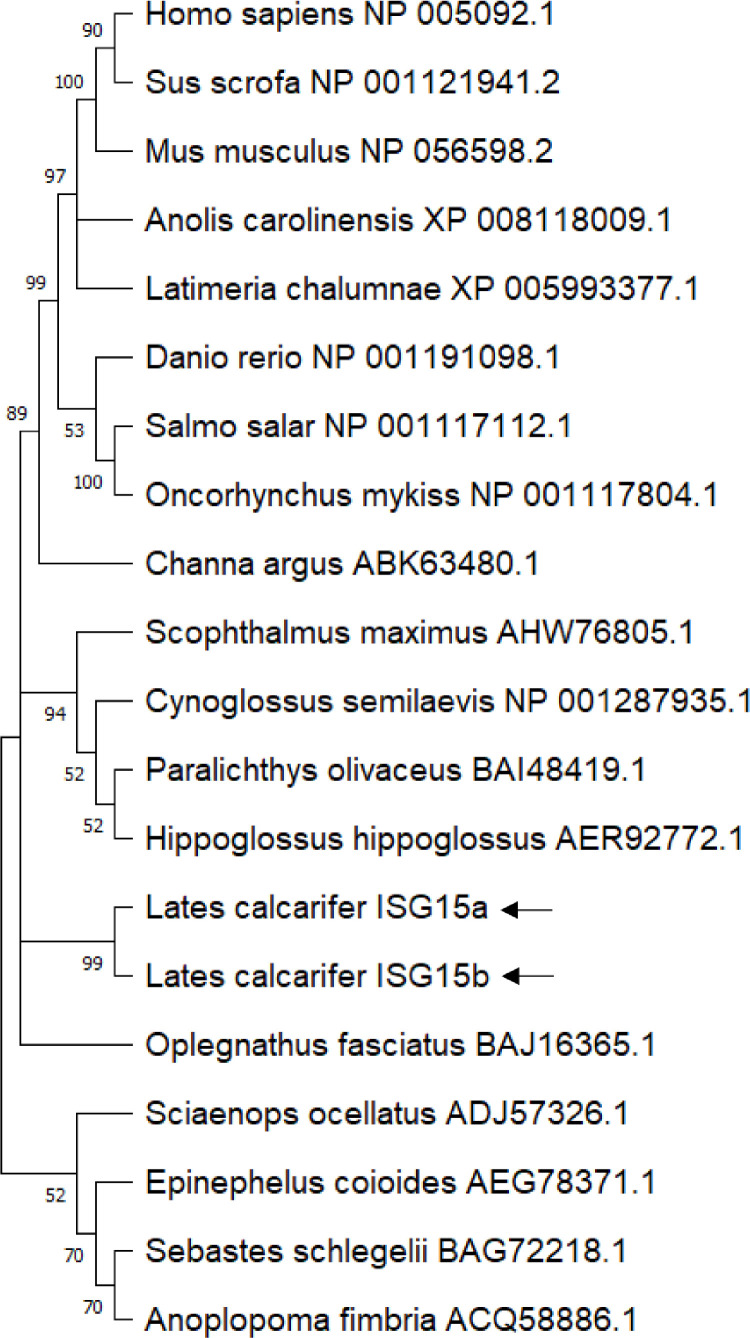
**Phylogenetic relationship of *Lates* ISG15s with other vertebrate ISG15:** We used the Neighbour-Joining method to infer the evolutionary history of the various ISG15 protein sequences. The percentage of replicate trees for which taxa are clustered together in the bootstrap test (500 replicates), is displayed next to each branch. Branches are drawn to scale, using the same units for branch lengths for evolutionary distances used to estimate phylogenetic distances. To calculate the evolutionary distances, the Dayhoff model was used. Nodes with lower bootstrap support (< 50%) in the phylogenetic tree were collapsed to polytomies. MEGA 11 software was used for the phylogenetic analysis. The *Lates* ISG15 sequences are marked by arrows.

Phylogenetically, the *Lates* ISG15A and ISG15B are grouped together and form a separate cluster from the other two perciform ISG15s – *Oplegnathus fasciatus* and *Sciaenops ocellatus*. The ISG15 homologues from the superorder *Acanthopterygii* including *Lates* ISG15 fall into a distinct group, whereas zebrafish (superorder *Ostariophysi*); salmon and trout (Superorder *Protacanthopterygii*) form different clusters. ISG15 homologues from *Latimeria* (Sarcopterygii) and the lizard *Anolis* (Reptilian) stood distinctly separate from fish and mammalian ISG15 (Fig. 3).

### 3.3 Identification of two *Lates ISG15* genes

**Fig. 4:**
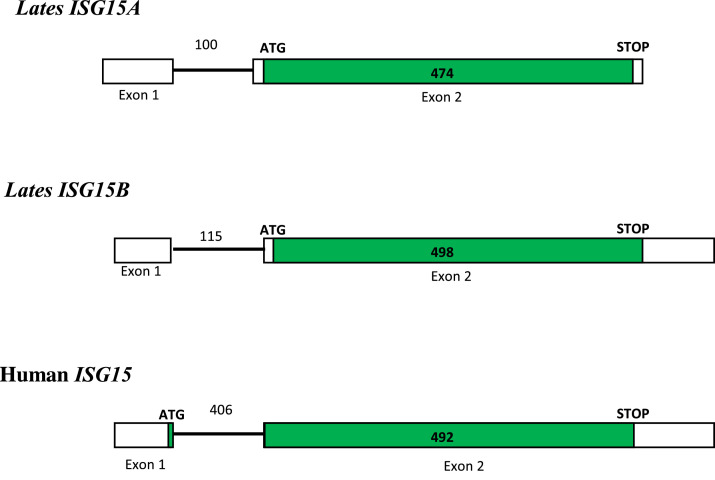
**Genomic structure of the *Lates calcarifer ISG15A* and *ISG15B* genes and comparison with Human *ISG15*.** Exons are represented by boxes and introns by lines. The green-colored boxes represent ISG15 protein-coding sequence. ISG15 gene is composed of 2 exons separated by an intron. In *Lates ISG15A* and *ISG15B* genes, Exon 1 does not code for protein, whereas the entire coding sequence is contained in Exon 2. In the human *ISG15* gene, ATG is present in the Exon 1 in human ISG15 and the rest of the protein-coding sequence in Exon 2.

The *Lates ISG15A* and *ISG15B* genes were amplified from genomic DNA using the designed primers (Table1). Both the genes are bi-exonic. The cloned *ISG15A* gene is 693 bp with a 100 bp intron separating exon 1 and exon 2. The cloned *ISG15B* gene (767 bp) has a 115bp intron separating the two exons (Fig. 4). We aligned the *ISG15A* and *ISG15B* gene sequences against the *Lates* genome and found that these two genes are located very close and separated by only 2.9 kb in the genome contig (Supplementary Fig. 1).

### 3.4 Comparative normal mode analysis of the dynamics of LcISG15A and LcISG15B

**Fig. 5:**
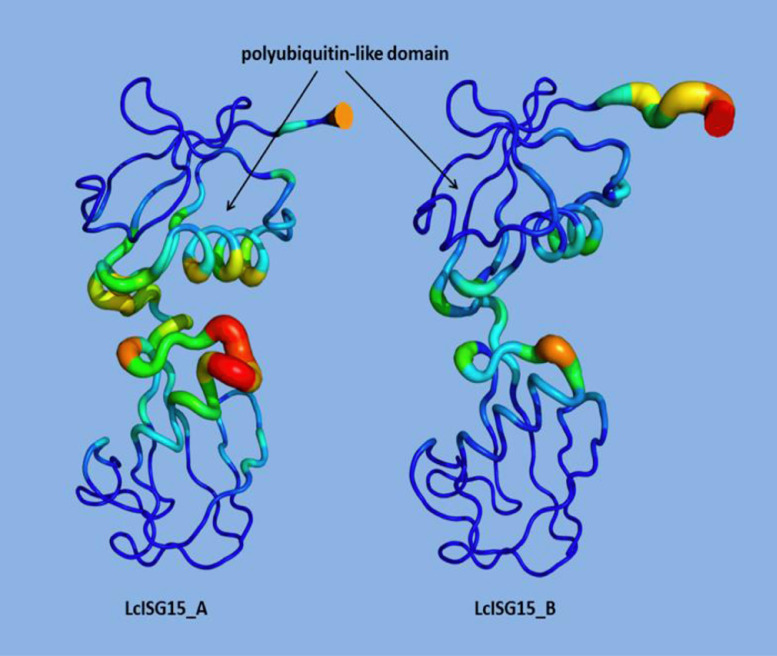
Predicted structures of LcISG15A and LcISG15B proteins are drawn in cartoon putty representation at intradomain linker regions, where the colour is ramped by residue from blue as the lowest deformation area to red as the highest deformation area. The size of the tube also reflects the amount of deformation, were greater the deformation, the thicker the tube.

LcISG15A and LcISG15B proteins share a similar β-grasp fold that has two ubiquitin-like domains separated by an intradomain linker. The predicted models of the homologues were built using the crystal structure of ubiquitin-like protein ISG15 (PDB:6bi8) from the Protein Data Bank (PDB) as the template for modelling. Understanding the dynamics of the isoforms is a useful method for the systematic investigation of protein folding and structure-function relationships [24]. Here, normal mode analysis based on elastic network models was used to determine the extent to which structural flexibility affects the dynamics of LcISG15A and LcISG15B, respectively. Although LcISG15A and LcISG15B appear very similar, the differences in light of their dynamics are explained. A diagrammatic representation of the homologues is given in Fig. 5. LcISG15A shows a significant deformation in the intradomain linker region absent in LcISG15B, concerning the atomic fluctuations in the residues positioned there. This deformation indicates that the movement of domains (I and II) changes significantly in LcISG15A, allowing for various functions or more opportunities to interact with other proteins or small molecules for additional functions that are unique between the ubiquitin and ubiquitin modifiers. However, a lower deformation predicted in intradomain residues reveals the lesser movement of domains (I and II) in LcISG15B with limited functions for substrate binding and catalysis.

### 3.5 Tissue expression of *Lates ISG15A* and *ISG15B* mRNA in healthy fish

**Fig. 6:**
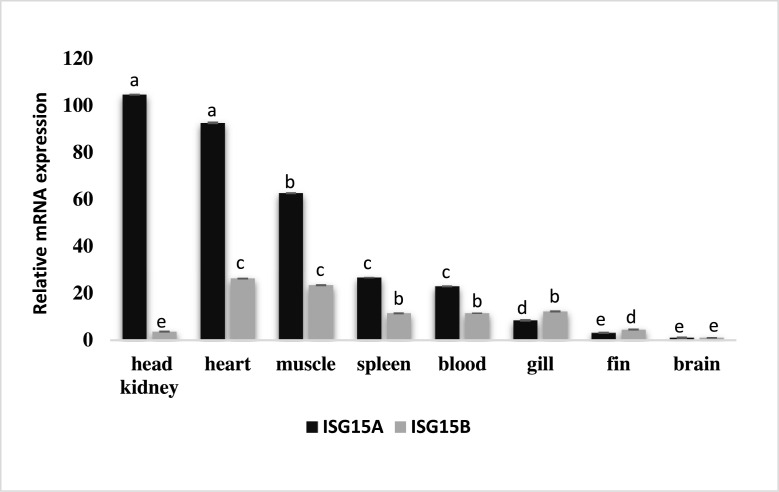
**Tissue expression profile of *LcISG15A* and *LcISG15B* in healthy fish.** The relative mRNA expression levels were normalized to beta-actin using 2^−ΔΔCT^ method and were calibrated against gene expression levels in the brain. The tissues were ordered according to relative expression levels from highest to lowest. Error bar represents the standard error of the mean, p < 0.05. The different letters denote statistically significant differences among the groups and within each group over the time according to Tukey post-hoc test (p < 0.05).

Tissue level expression profile of *Lates* ISG15 gene homologues reveals that both genes are ubiquitously expressed in all tested organs like spleen, head kidney, gill, brain, blood, fin, heart and muscle. The expression level of LcISG15A mRNA was more in the head kidney (104-fold), followed by heart (94-fold) and muscle (62-fold) were as LcISG15B mRNA is expressed more in the heart (26-fold), muscle (23-fold) followed by spleen and blood (11-fold) (Fig. 6).

### 3.6 Expression profile of *Lates ISG15A* and *ISG15B* mRNA upon poly(I:C) and RGNNV challenge

The expression pattern of LcISG15 homologues in different tissues like head-kidney, gill, spleen and heart upon poly(I:C) and RGNNV challenge was investigated. Both LcISG15A and LcISG15B expression was upregulated and peaked at 6 hours of post-challenge in all examined tissues following poly(I:C) challenge. In the head kidney, ISG15A showed a 196-fold increase and ISG15B showed a 381-fold increase at 6 hours post-challenge. In gill, ISG15A showed a 224-fold increase and ISG15B showed 13.5-fold increase at 6 hours after challenge and gradually decreased. In heart, ISG15A showed a 523-fold increase and ISG15B showed 364-fold increase at 6 hours post poly(I:C) challenge. In the spleen, ISG15A expression was 213-fold and ISG15B expression was 24.9-fold at 6 hours post-challenge and gradually decreased (Fig. 7a).

**Fig. 7a:**
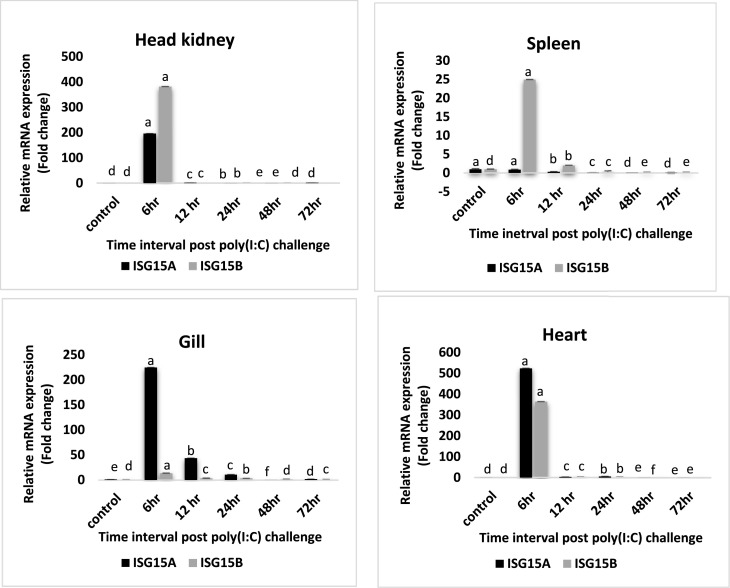
LcISG15 homologues expression in different tissues (head kidney, gill, spleen and heart) after poly(I:C) challenge. The relative expression levels of LcISG15A and LcISG15B were normalized by beta-actin and expressed as fold changes by comparing the normalized gene expression levels of poly(I:C) treated fish with that of PBS injected fish at the same time point. Error bar represents the standard error of the mean [mean ± SD (n = 3)], p < 0.05. The different letters denote statistically significant differences among the groups and within each group over time according to Tukey post-hoc test (p < 0.05).

Following the RGNNV challenge, there was a significant increase in the expression of ISG15 in the head-kidney, gill, spleen and heart. In the head kidney, the expression of ISG15A and ISG15B reached the maximum at 12 hours post-challenge (4.4-fold and 12.5-fold respectively) and then gradually decreased. In gill, ISG15A expression reached a maximum at 6 hours of post-challenge (4.6-fold), and for ISG15B the highest level of expression was seen at 48 hours of post-challenge (34.9-fold). In the heart, ISG15A and ISG15B gene expression reached a maximum at 24 hours of post-challenge (49.96-fold and 9.66-fold, respectively). In the spleen, both ISG15A and ISG15B showed two peaks; one at 6 hours of post-challenge (3.7-fold and 15.7-fold respectively) and another at 24 hours of post-viral challenge (2.5-fold and 17.38-fold respectively) and decreased gradually (Fig. 7b).

**Fig. 7b:**
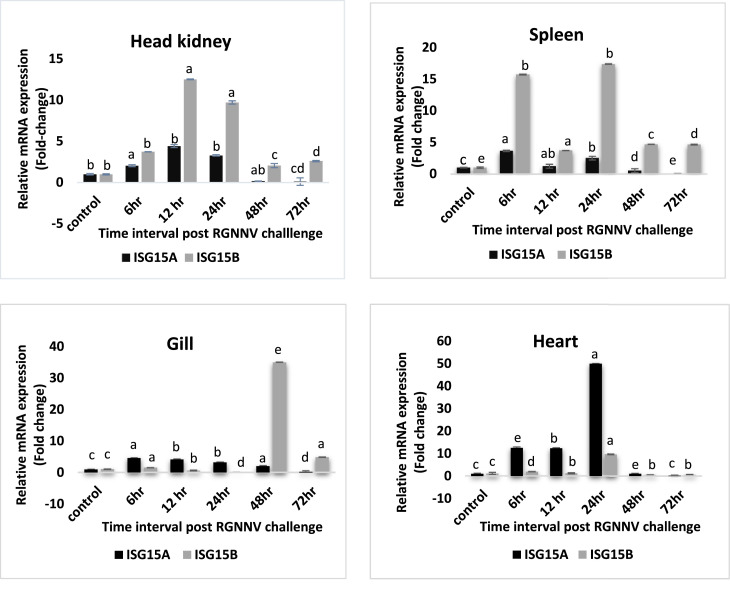
LcISG15 homologues expression in different tissues (head kidney, gill, spleen and heart) after RGNNV challenge. The relative expression levels of LcISG15A and LcISG15B were normalized by beta-actin and expressed as fold changes by comparing the normalized gene expression levels of RGNNV injected fish with that of PBS injected fish at the same time point. Error bar represents the standard error of the mean [mean ± SD (n = 3)], p < 0.05. The different letters denote statistically significant differences among the groups and within each group over the time according to Tukey post-hoc test (p < 0.05).

## 4 Discussion

Antiviral immune response in fish involves the expression of type I IFN and subsequent transcription of numerous interferon-stimulated genes (ISGs) that lead to an antiviral state. The interferon-stimulated gene 15 (ISG15) is one among the early and highly expressed ISGs upon viral infection. It is a 15 kDa effector protein with a proven antiviral role [10]. In most fishes, the genome contains only one homolog of the ISG15 gene. The presence of more than one homologue of ISG15 in fishes is rare and was reported in a few fishes like Atlantic cod with three ISG15 homologues, crucian carp and large yellow croaker with two ISG15 homologues [17,30]. This study reports the identification, characterisation, and expression analysis of two homologues of ISG15 from *Lates calcarifer.*

In human and other mammalian ISG15s, the two UBL domains are separated by a short five amino acid linker or a hinge region. A conserved cysteine residue in this hinge region is reported to be involved in the ISGylation of target proteins. This residue (Cys-78) in the hinge region of ISG15 interacts via a disulfide bridge to the substrate proteins to facilitate the post-translational modification [31]. In human ISG15, the Cys-78 is the only cysteine residue in the ISG15 molecule. Interestingly *Lates* ISG15 and other fish homologues do not have this conserved cysteine residue, as it is replaced by proline. LcISG15A has two Cysteines (Cys-114 & Cys-151), whereas LcISG15B has only one at Cys-149. The LcISG15A Cys-151 and LcISG15B Cys-149 seem to be homologous based on the amino acid alignment, located just before the RLRGG motif near the C terminal (Fig. 2a & 2b). The cysteine residues in ISG15 do not seem to be strictly conserved, unlike the cysteines in the IFN mature protein sequences. There is only one cysteine residue in *Salmo* ISG15 (Cys-133) and *Sciaenops* (Cys-61), *Oplegnathus* ISG15 has two (Cys-13 and Cys-113), and the latter seems to be homologous to the *Lates* ISG15A Cys-114 (Fig. 2b). Zebrafish ISG15 does not seem to contain any cysteine residues. The role of cysteines in ISGylation among the various piscine ISG15 would be worth investigating.

*ISG15* gene is believed to have originated by duplication of the ubiquitin dimer and insertion into an IFN controlled environment. Ubiquitin is one of the most conserved polypeptides found in all eukaryotes comprising 76 amino acids. Unlike ubiquitin, ISG15 does not seem to have a very high level of sequence identity between species [32]. Both *Lates* ISG15A and ISG15B proteins show overall structural conservation with two tandem UbL domains linked by a short hinge region. The first UbL domain was identical in both LcISG15 homologues and has only 27% amino acid identity and 51% similarity with the 76 amino-acid Ubiquitin protein. The second UbL domain was different in LcISG15A and LcISG15B, with only 62% sequence identity between them. The second UbL domain featured the characteristic LRLRGG sequences and had a higher identity (44%) and similarity (67%) with Ubiquitin protein than the first UbL domain (Supplementary Fig. 2). The C-terminal or conjugating domain of ISG15 is involved in covalent bonding with E substrates by its C-terminal LRLRGG motif. In contrast, the N-terminal domain has a regulatory role in enhancing the E3-mediated transfer of ISG15 from conjugating enzyme UbcH8 to ISG15 substrates [33].

A high degree of conservation of the UBL domains, especially of the six aliphatic residues and the canonical C-terminal amino acid motif LRLRGG that are crucial for the ubiquitin-like protein structure [34], was observed when deduced amino acid sequence of LcISG15A and LcISG15B were compared with other piscine and mammalian ISG15 (Fig. 2a). Mutations of residues in the LRLRGG motif abolish the ISGylation of cellular and viral proteins critical for antiviral activity in fish ISG15 [34]. Most of the fish ISG15 lack any C-terminal extension beyond the LRLRGG motif, and such extensions were reported in only a few ISG15 orthologs from *Paralichthys olivaceous, Sciaenops ocellatus, Solea senegalensis, Cynoglossus semilaevis and Larimichthys crocea* [17,35]*.* The presence of two homologues of ISG15, one ending with LRLRGG motif and another with a C-terminal extension beyond the LRLRGG motif, seems to be unique to *Lates calcarifer*. Only a few mammalian ISG15 proteins like human, mouse and rat ISG15 are translated as a precursor with 5-8 amino acid extensions beyond the LRLRGG motif [36]. Reports suggest that ISG15 in humans and mice are synthesised as a precursor protein post-translationally processed by a cellular converting enzyme that cleaves additional C-terminal amino acids, exposing the LRGG residues for conjugation. The lack of these additional amino acids on LcISG15A could allow the exposure of the conjugating motif and hence suggest that post-translational processing is not required. However, its homolog LcISG15B that has additional amino acids may undergo post-translational modifications to become an active protein. It has been reported that the translation of these Ubiquitin-like modifier proteins with an extended C-terminal prevents direct conjugation with the target proteins [37].

The alignment of cloned *Lates ISG15A* and *ISG15B* genes with the genome revealed that the two genes are located close in the genome. The gene structure of both the *Lates ISG15* genes is typical of other fish homologues with two exons and a single intron flanked by the canonical GT /AG splicing motifs (Supplementary Fig. 1). The intron region is located at 5′ UTR of the *ISG15* gene as reported in other teleost fishes suggesting its role in regulating the ISG15 protein level [38]. Reports suggest that the maturation pathway and translational efficiency of mRNA depend upon the position of the intron. The mammalian *ISG15* gene has a single intron within the ORF after first methionine, whereas intron in piscine *ISG15* was reported at the 5′ UTR. The presence of an intron at 5′ end of ORF may direct mRNA toward translational silencing, and the one at 3′ end represses the translation [39]. *Lates ISG15* gene homologues are also intronless in the ORF like other teleost *ISG15*s, suggesting translational silencing. The introns in *Lates ISG15* (100 and 115 bp) are shorter when compared to human and mouse orthologs which are almost four times larger (492 and 483 bp). The European seabass (*Dicentrarchus labrax*) *ISG15* was reported to have an intron twice the size of *Lates ISG15* (276 bp), whereas cod, flounder and zebrafish have comparable size [24]. Interestingly, only the second exon in both the *Lates ISG15A* and *ISG15B* code for proteins (Fig. 4). This is in contrast with the human *ISG15*, where the ATG is contained in exon 1 and the rest of protein in exon 2. Both the *Lates ISG15* genes have IFN-stimulatory response elements (ISRE) located around 250 to 280 bp upstream of the start codon at the promoter region. These ISRE elements along with transcription factors in IFN signalling like IRF3, regulate ISG15 expression in response to IFN or viral infection [40].

The putative 3′ UTR was predicted from the genome by identifying the AATAAA polyadenylation signal downstream of the stop codon. The instability motifs or sequences that affect mRNA stability and translation efficiency were reported from several fishes varying from one in zebrafish (*Danio rerio*), four in European seabass (*Dicentrarchus labrax*), six in Japanese flounder (*Paralichthys olivaceus*) ISG15s. In contrast, no instability motifs were reported from mammals such as *Homo sapiens, Mus musculus or Bos taurus* [24,30,41]. In our study, one instability motif (ATTTA) was identified in the 3′ UTR of *Lates ISG15A* mRNA, whereas two such motifs were present in *Lates ISG15B.* The Cytoplasmic Polyadenylation Element (CPE) that are U-rich sequence able to repress or exert translation depending on the cellular type [42] was found only in the *LcISG15B* 3′-UTR region (Supplementary Fig. 1). This motif is also present in several other fish ISG15, such as zebrafish, Japanese flounder, turbot, or European seabass [24]. Hence it may be presumed that the *LcISG15A* and *LcISG15B* may undergo different post-transcriptional regulations as well. The presence of both types of regulatory elements in fish but not in mammalian ISG15, suggests that the expression of fish *ISG15* genes are subjected to tight post-transcriptional regulation.

*Lates ISG15A* and *ISG15B* mRNA were ubiquitously expressed in all examined organs. The *Lates ISG15A* was predominantly expressed in the head kidney, heart, muscle and blood. *LcISG15B* expression was more in the heart, muscle, spleen and blood (Fig. 6). Tissue level expression studies of *ISG15* gene in other teleost fishes like *Sebastes schlegelii* [43], *Paralichthys olivaceus* [44], *Cynoglossus semilaevis* [45], *Epinephelus coioides* [46], *Scophthalmus maximus* [30], *Solea senegalensis* [35] and *Larimichthys crocea* [17] have indicated that *ISG15* was constitutively expressed. In fishes like *Cynoglossus semilaevis and Solea senegalensis, ISG15* gene expression was high in the heart [35,45]. In *Epinephelus coioides* and *Scophthalmus maximus*, the head kidney showed the highest *ISG15* gene expression. The expression of *ISG15* gene in different tissues suggests that they have a diverse functional role in translation, splicing, cytoskeleton organisation, stress response and chromatin remodelling [47].

Polyinosinic: polycytidylic acid (poly I:C) is a double-stranded RNA (dsRNA) that mimics viral Pathogen-associated molecular patterns (PAMP). They bind to TLR3 and signals to activate an extracellular dsRNA-activated antiviral pathway or bind to RIG-I/ MDA5 and leads to translocation of NF-kB to the nucleus that results in transcription of interferon gene and results in an antiviral state. In our studies, *Lates calcarifer* injected with poly(I:C) showed an early and rapid induction of ISG15 mRNA at 6 hours of post-challenge, indicating its antiviral role. Our results give evidence that among the two homologues, LcISG15A showed a rapid upregulation when compared to LcISG15B following poly(I:C) challenge. The lack of any C-terminal extensions beyond the LRGG domain may give ISG15A a rapid and enhanced capability of interacting with substrates without additional post-translational modification. *ISG15* gene expression showed similar expression in early hours as reported in Atlantic cod ISG15 homologues [41]. Other reports show significant upregulation of ISG15 gene expression in *Dicentrarchus labrax, Solea senegalensis, Scophthalmus maximus, Oplegnathus fasciatus, Gadus morhua, Paralichthys olivaceus, Larimichthys crocea* after poly(I:C) challenge [16, 23,29,34]. Among the various tissues tested, the leukocyte-rich organs of *Lates* such as head kidney, heart, spleen, and gill showed higher levels of ISG15 basal expression (Fig. 6), and upon poly (I:C) injections ISG15 transcripts were highly induced (Fig. 7a). A similar pattern of ISG15 production has been reported in *Solea senegalensis* and is attributed to the production of ISG15 by leukocytes [35].

The red-spotted grouper nervous necrosis virus (RGNNV) challenge causes an upregulation in ISG15 expression in the head kidney, spleen, heart and gill of *Lates calcarifer*. The upregulation of the expression of the LcISG15 homologues upon virus infection may give evidence about the active roles of these genes in antiviral response. In *Lates,* poly(I:C) challenge gave an early expression of both ISG15 homologues compared with that challenged with RGNNV. Poly(I:C) is a concentrated and pure PAMP that can activate the host immune response strongly and quickly by inducing type I IFN and ISGs [48]. But RGNNV has to go through replication cycles to multiply and trigger detectable host immune responses. Viruses have evolved elaborate mechanisms to suppress the host immune system, especially the interferon system, and nodaviruses like RGNNV are no exception. The B2 protein of RGNNV is known to negatively regulate the host IFN response, accompanied by suppression of several ISG genes, including ISG15 [49].

Studies in other teleost fishes have also demonstrated that ISG15 plays a key role in the innate immune response against other piscine viruses threatening commercial aquaculture systems. For example, in Atlantic salmon against Infectious Pancreatic Necrosis Virus (IPNV)[50], in *Scophthalmus maximus* against Turbot reddish body iridovirus (TRBIV) [30], *Solea senegalensis* and *Dicentrarchus labrax* against NNV (Noda virus) [24,35], in *Paralichthys olivaceus* against Hirame rhabdovirus (HIRRV) [44].

RGNNV challenge studies in *Lates* heart and spleen showed an upregulation of *LcISG15A* at 6 hours of post-challenge. Similar results were reported on grouper (*Epinephelus coioides*) ISG15 against GNNV infection, where it showed a rapid upregulation than poly(I:C) treated one [46]. In *Lates* spleen, ISG15A and ISG15B expression showed two peaks, one at 6 hours and the second at 24 hours of post-challenge. Similarly, two peaks were reported in turbot ISG15. It may be due to the activation of both IFN-dependent and IFN-independent pathways. The first expression peak may be directly activated by virus stimulation which is IFN- independent. It directly stimulates ISG15 by binding Interferon Response Factors (IRFs) to ISRE elements in the ISG15 gene promoter and doesn't involve the synthesis of any new protein. The second peak is IFN dependent in which ISG15 is produced as an inducible product of type I IFN signalling cascade upon viral infection. Due to additional protein signalling activation cascades involved in the IFN-dependent pathway, the second expression peak is delayed. A similar pattern of gene expression was reported in turbot following TRBIV challenge [30].

In this study, we have only used one virus - RGNNV, to evaluate the role of ISG15 in antiviral response in Asian seabass. The role of ISG15 homologues would also be worth investigating in response to other major viral pathogens of *Lates* like Infectious Spleen and Kidney Necrosis Virus (ISKNV), Lates calcarifer Birnavirus (LCBV), Lates calcarifer herpes virus (LCHV), Scale Drop Disease Virus (SDDV) etc. The availability of *Lates* kidney cell lines like SISK and real-time PCR primers for *Lates* ISG15 gene expression enable cell-based experiments to be developed to understand the role ISG15 in the host immune response against these pathogens or to evaluate potential antiviral molecules *in-vitro*.

In summary, we identified the two homologues of the *ISG15* gene from *Lates calcarifer; ISG15A* and *ISG15B*. These genes reside close together in the genome and share a similar overall structure to other vertebrate *ISG15* orthologues. However, these genes and their gene products seem to be functionally different, considering their second ubiquitin-like domains and the C-terminal extension after the LRGG motif. Tissue level expression studies reveal that these genes are functionally significant with ubiquitous expression in all examined tissues. The *LcISG15* homologues are upregulated upon challenge with poly(I:C) and RGNNV suggests their role in antiviral defence. Further studies on *Lates* ISG15 in other viral infections will provide a deeper insight into innate immune antiviral mechanisms in *Lates calcarifer*, which may lead to the development of new prophylactic strategies and immunological tools to improve fish health.

## Declaration of Competing Interest

The authors declare no known competing financial interests or personal relationships that could have influenced the work reported in this manuscript.

## Appendix Supplementary materials

Image, application 1

Image, application 2
