# Supplementary material for: Identification of two ISG15 homologues involved in host immune response against RGNNV in Asian seabass (Lates calcarifer)
Source: Fish Shellfish Immunol Rep. 2022 Mar 8;3:100054. doi: 10.1016/j.fsirep.2022.100054 (PMC9680060; doi:10.1016/j.fsirep.2022.100054)
Supplement: Supplementary file 1 [file mmc1.docx]

ACAGTGGAAGAATATTTTGTGGCACCACCAGGTGAGAAAAAAGCCTGAATATAACAATATCGCTGATTTAGTGATTTATTGTTCACACCTGTTTCTGTAACTGTGAGGATTCTTAACTAAGATTTTTTTTTTACCACAGGAAATATTCCTTTACCAAAGAGGGAGGAGAGGGCTGCCATACTGAAACACTCAGAGCTCTGATATTTATGAATTTGTCCTCTGAATTTCTTACCAGTGCTTTTATTTACAGACTGGATGTGTCTTAGAAGAAAGTATGTGTCAGATCTGTTGGCTGAGGACAAGACTTGGTCACTTCACTGCTGATCTGTCAGGAAGCTTTGTCAGACAGTACAGGAAAAATAAAAAAGCTGCAGGTCATTTATGTTCATTTTTCAGTTTCACTTAACACCTGTGGTTAAATGTTTTTGTTTAGTTTCACTACCAGTTCATAAAAATGGGGAGTGAACAGTGTGCCAGGGCAGAGGCTGTGGTCAGGCTATTCAGTGCAATTGTTTTGATTTGTGATTTCCATTATTATTTTCATGTGTCTTGTTTTTTTAACTAATAAAAAAAGAAATCCTTAATCTGTTCTGTGTGTGTGTGTCTGTATTGTTACCATAACGTTTTTTGAAGGATACACAGTCGCAGTTCATCTGATGAAATAGTTACCTCTGTGCGTGTAATGTTGGGGGAGCGTTTCTTTGACGGCCCAGTGTTCAAACCGTTCATTCTTTCCTTACTCGAACTACACAGCCCTTGTTTGACCTTTTCATTTTCTGTTTCCCTGTTTGTTTGTATCATTAGCTAATAAATTCATAAACCTGCCGCGGGGTTTGCCATTATCAAAACATTACAAGCCTGCATTACAGCAGAGTCATGTTCGAGAG**GAAACCGAAA**GTTATGAAGGCAGCGGAGG**GAAAACGAA**TCTTACGTGAGTGGGAGGGAGTCGATCAGCTGAGACAGCAGCTGTA**TATAAA**CTGCACTGAGCTCAGACCTAAAACACAGCTGCTGCAGAGCATCGTCTGTTTGCCTTTCGCCGTTTGAAGACTTAAAATTTCCGGAGAAGTTTGTTGTTGTTTAGTGTCAAAAAG**GT**AAGAAACTCTCTTAAACAACAGTGTGGTGCGATACACTGTATTCTGCGAGTTTTTCTACAGTAGACTAAATCTAAAAATGTTTTGAATTGTCTTTC**AG**ACCACTTTCAAC**ATGGATATAATCATCACTATGCTGAGTGAGACCCGGACAGTGAGGGTTCACGCACAGGCGACAGTTGGAGATCTGAAAAAAATCATCCACGAGAAGCTGGGAGTCCCCCCTCAGAGACAGAAGCTGGTTTTTGTCAACGGTCAGAGGACGGATCTCAGCAACGATTCACAGCCCATCACCTACTACGGCGTTCAGTCTGGATCCAGGGTGTCCCTGCTGGTGACCGAGCCGACCCAGCCGGCGACCATCCAGGTGTTCCTGAGGAACGAAAAGGGGCAGTTAAACACCTACGACATCAAACCTGACGAGACAGTCAGTAACTTCAAGAGCAAGGTCCAGTGCAGAGAGGGGGTGCAGGTGAGCCAGCAGAGGCTCGTTTTCCAAGGCCGGGAGATGAGTGGAGGTTACCTGTCTGACTACAACGTCCAGGCGCTGAGCACCATCGACTTGTGTCTCCGTCTGAGAGGAGGCTGA**GGACCCTGTCACACGGTCACTGTTAGAAACTAATGGAGACGCTTATCTA**ATTTA**AATACTGATCAAATCATCCCTCG**AATAAA**ATGTTTGAATCTTTAAGCTTCACTGTATAGCCCATGTGGAGAGTTTGACGATATATTTACTGATCAAAGTGTACATTTTCTCTGTGCTCAGTGAAAATCTGACTTTAAGAGGCGGACCCATGATGTCTTGAAAGAGAGTCCTGCTCCAGTGTTGAGCGAGTATGAGTTTGAAGTTGAAGTCTACACTGAGGTTGGACTTTACAGTGAAGTATGAGTCACCTGGTTTCCGGAAGTTAAACCTGTCAGATGAAAAATACTTTACACAAAAGTAAAACATATTAGAGTATTTATGTATGTCTTGTGGAGGGGACCTTTTAATGTCCTATTTATTTTTTAGCTTTATTTTGAGAAGAAAAAAAACAAGCAATGCATTCCTCACTGTGTAGCACTGCCATATGTCAAAAACTAATGATGCTAATTTTGTTTTGACCCTACAGATATATCATATACACAGCACAACAGATTTTGATAAATACATGTTTCAATCATGGTATGGCAGATTTTGTTTTGGTTTGATATGTAATTTTTTATTCTGGAAATACAGGTATAATTGTTGAGTATTGAATAGACAAATACAACACTGTGTTAACAGTTCACTGTAAAAACTGAAATAAAGTCACAAATGTGAATTTACATGTTTCCTCTCATTTCCTGTTCTTGTTTTGAGCTCAGCAGTGGCGCTGACACCCTGTACAAACCACAACATCTCAGGTCTCTGGCATTAACAAGTAGACACTTTAGTCATCATTAAGTCAAACAAACATGACTGTACTGTTACTTTTGAATTTGTGTGAGCGCAACAGGAAAGTCATGACAAAATTACCTTTAGATGTTTAGTAAACATCATGATTTTAAATCGAGGCTCCTACTAAACAACTGAGAAACACTAACAAACTGAATTATTTCACAGTGGTTTATTAGAGCATGGTAAGTAAAATATTTAGCACAACTCAGCATGAAATATCACATGTACATAGTCAAACATGTTTTATAAGACACTCAAAGAAGCTTGACTACACAATATTTTCAGTCTGCCTCCCTTTTCTTGCATAGCCTGTGAAAAAACTAATACATACTCCTTGTCAGAGGACCCCCATAGAAGACAGTGGTTGAATAAATATCAAATACAAATTATATTTTAAATTATGTGGCTAATTGAGAGCCTCACTACCTCAGATGTTATGGTTCACTTAATTCAGTCTGAACAACAACATGCCATTAGCTATCAAGGTTTGCGTTTATCTTTGGTTGGGGCATTTCCTTGTCGGACAGCCAAAATAAAAAGTTGTGTGTGTCCTGTTACTCTCACACACATGCATATGAATGATAGAGTCACAGCAGCTTTAACACATTTGTGTCTCTGTCTCTTGCTCAGGCTGCTCCTAGCGGGATTTTCAGAATAAAATGATGCCTAATAATAACAAACAAAACCTGGACAAGCTGTGGTTAATTCACCTTATAAATGTAATAATATGTTCATGTCTTAATGACCTATATGCAGTAGGCTAATAAATCTGTCCCTGATCCAGGGATGGCATTAAATAGTAAATATTTCTCCCACTAGTTTTGGAGAAGATATATCTTCTATGGCCTTGGATTTTAATAATACAGATGTCCTGCACAGAGGAAGAGTAAAAATCATCAGGTGTCCAGTCTGACTATGTGTTCGTGTTTTCACTGGTCAGGCTGCATAATAAATGGATGATTCATCAGCAGGTCCTGTATAACATTATCTCAGGAGTTTCATGAAGCTGCAGGTGTAGAGTCCCAGGGGTGTTGGTTTGTTGTGTCTTTAATGAGTTGTGTTTCTGTACGTCATAGTGTGCTTAGTCAATCATTTAGTTATCAGCAGTGATTGTAATGTGAAATGGACTGTCAAAATTTGTTTGGCAAGAAATTCTGTGAATCATGATGCAACAGGTACGGCATCTCCTCACGACATTTAAAATCAGTTAATGCCTGAATCCTGCCCAGAGCATTGTGCTGAAATAAAACAGAAATAGCACTTTTAATCAGTAATGTATACCTTAATTGGAAGAGCTGAAAAAAATCATGGACTTCCTGGGGTGGGGGTATGTTAGGGTTTGTAAACAGTGATGCTGCTAGATGAAAGAACAGGAAGAGAGAAACAAAACTCTCTAGGAGCAGAGCAGAGTTTCACTGTAAATAAATAAAGCTCAGCACATTCAGGCCTTTACAGCCGAGGGTTAGCTGAGGTCAACACTTGGTCACTTCTCTGCTGATCTGCAGCCTAAAACAGCTGTCAAAACACTTTGTCAGATATAAAGAACAGGAAAAATAAAAAGCTGCTTGTCATTTATATTCACTTTTCAGTTTCATTTAGCACCTGTGGTCAAAGCTTTTGTTTCAGTATCAGTTCATAAAAATGGTGAATGAGCAGTGTGCTGATGCAAGAGAATGTAGTGCAGTGGCAGTACATTGTTTTGAATGGTGATTAACATCATTGCTTTTCACTGACTTTAATAAAATTTTATTCTCTCTCTTTCTGTTTTGTTTCCGTAGCAAAACGAATAGTTAAAAATAGGCTCTGACGTGCCTGTGCTGTCAGTCACATGCATCTGATGAAATGATTGTCTTTTCTTATTGACAGCCCTGATTATGATCTTACATAACTATGTAAACGTCCCGTCCTGTAATGTTTCTTGCAATTCCCACTAACAACTCTCTGTGCTCTGACTCGTCTGTTTGTTTAAAACTGTGCTACTAGGTGGTAAATTCCTACATACGCCTCGGGCAGTGTTATGCTGATCACAACATTACAAGCCTTCATGACAGCACAGTTATGTCAGAATA**GAAACCGAAAG**TTATCAAGGCAGAGAAGG**GAAAACGAA**TCTGTAAGTGGGGTGGGAGGGAGTTGATCAGCTGAGACAGCCTGGA**TATAA**ACAGCCGAGTCAACCTTCAAGCGCAGCATTGCCTGAGCTGAAGACTTGAAGAGGAGTTTGCTGTTTAGTATCAGAAG**GT**AGGAAACTCTCAAAACACACACACACACACAGAGGGACACCGAGATAATTCATTGCAAATGTTTTTTTTTCTACAGTAGACTTATTCTAAAAATGTTTTGAATTGTCTTTC**AG**ACCACTTTCAAC**ATG**GATATAATCATCACTATGCTGAGTGAGACCCGGACAGTGAGGGTTCACGCACAGGCCACAGTTGGAGATCTGAAAAAAATCATCCACGAGAAGCTGGGAGTCCCCCCTCAGAGACAGAAGCTGGTTTTTGTCAACGGTCAGAGGACGGATCTCAGCAACGATTCACAGCCCATCACCTACTACGGCGTTCAGTCTGGATCCAGGGTGTCCCTGCTGGTGACCGAGCCGCAAGTCGTCCAGGTGTTCCTCAAAAACGACAAAGGGAATATTAATACCTATGATATCAAACCCGATGAGGCAGTCAGTAACTTCAAGCGCAGGGTCCAGAGCAGAGAGGGGGTGCCGACGGACCAGTTCTATCTGACTTATGAAGGCAAGCAGCTGATGGATGGTAGTAAACTGTCTGACTACAACATCGGGGAGATGAGCACCATCAACATGTGTCTGCGTCTGAGAGGAGGCTGGGGACACTCTGACATGGCTGAGATATCA**TAG**TAATAAGCACTAAATCAAGTTAATACCTATTGTTTT**ATTTA**AGTTACGTGAACACAAAGCACTCTGATCGTTAGTTTATTTTTAT**ATTTA**TTTTATTATTTCGAAAAATGAACAGCCTACTTTACTTAGGCTTTATGTGGTGTGATTCAGTTATGTGTTAAAAACATGTTTTGGTTCACTGTGTTACTTTGTATTCTGGCAATACAAGTGTGACTCTTAACATTTTTAACTGACAGCATGACATAACTATGTTAGCTGGGCACTGTAAAAACTGA**AATAAA**GCCACTAATGTCAATTAACTGTCTCCTGTACCTTTCTTAAGCTCAGTGGTGGAGCTCTACCCTGTACCACAACCACAGGAAGCAACCAAAAGAAGGCTTACTTAAGAATTGCATATTCAAAACAGACATTATTTCAATATTTAAAAAGAATTACTATAACATCTTAGAAGATTTTGGCAGTGCTCTATAAAAGAATACATTTATTATAAACATTTATTATGTTCTGGTATTATAAACACAGTCATGTTTGAAACATATCATAGCAGCATGATATAATGGCTTAAACTCATCGTAGAGGTTTGTTAATCAGAAGAGCTACTGTGTGTGAAAATTATTTTCCAAATTAGGAACAGGTTTTCAGTTGTGCATTAAACACTCTGGCATTCACAGGTGTGCAACAAAGCTGCTGTGAAGAAGGTAATTCCTCCAGATTTGTTGGAACATCATTTCCTCACTGACGCAAGGTAGGCATACCAAAACAAGGCGATCACGTTGGCAAACAGCACCCTGAACTGTAAGCAAAAACAAACAGGAGCGTTTGAAGTGTGGTTGGCATGGGACTCATCATTTGTTCAACAACAGGATGTTGGTGAAATGCATACCTGAACAGGCACAAAGTTGACATTGACGAATTGGAAGGGAGTCCAAACTTTCCAATTCATCTTCAAAGCAGTCCAGTAACTTCTCTTCATCTTCTTTTCAAAGTCTTCCCACCCTTTGGCCTGAAAACAGATTGAAACCACCTTATATTTAGATGAAGCCAGTGGACTAAATATTATGGCCGTATC

***ATTA motif***

**Poly adenylation signal**

**LcISG15a Intron**

**ISRE-1 ISRE-2**

**TATA signal**

**LcISG15a CDS**

**ISRE-1**

**ISRE-2**

**TATA signal**

**LcISG15b Intron**

**LcISG15b CDS**

***CPE***

***ATTA motifs***

**Poly adenylation signal**

**Supplementary Figure 1:** Identification of the two cloned *Lates ISG15A* and *ISG15B* genes inside the *Lates calcarifer* isolate ASB-BC8 unitig_5945_quiver, whole genome shotgun sequence NCBI Genbank Acc No: LLXD01000096.1. The open reading frames (ORF) of the LcISG15A and LcISG15B gene are marked in blue font with the start and stop codons highlighted. The introns of the two genes are marked in grey highlight and the characteristic 5’ GT and 3’ AG intronic splice motifs marked.
