# Supplementary material for: Identification of two ISG15 homologues involved in host immune response against RGNNV in Asian seabass (Lates calcarifer)
Source: Fish Shellfish Immunol Rep. 2022 Mar 8;3:100054. doi: 10.1016/j.fsirep.2022.100054 (PMC9680060; doi:10.1016/j.fsirep.2022.100054)
Supplement: Supplementary file 2 [file mmc2.docx]

**A) Conservation of Residues**


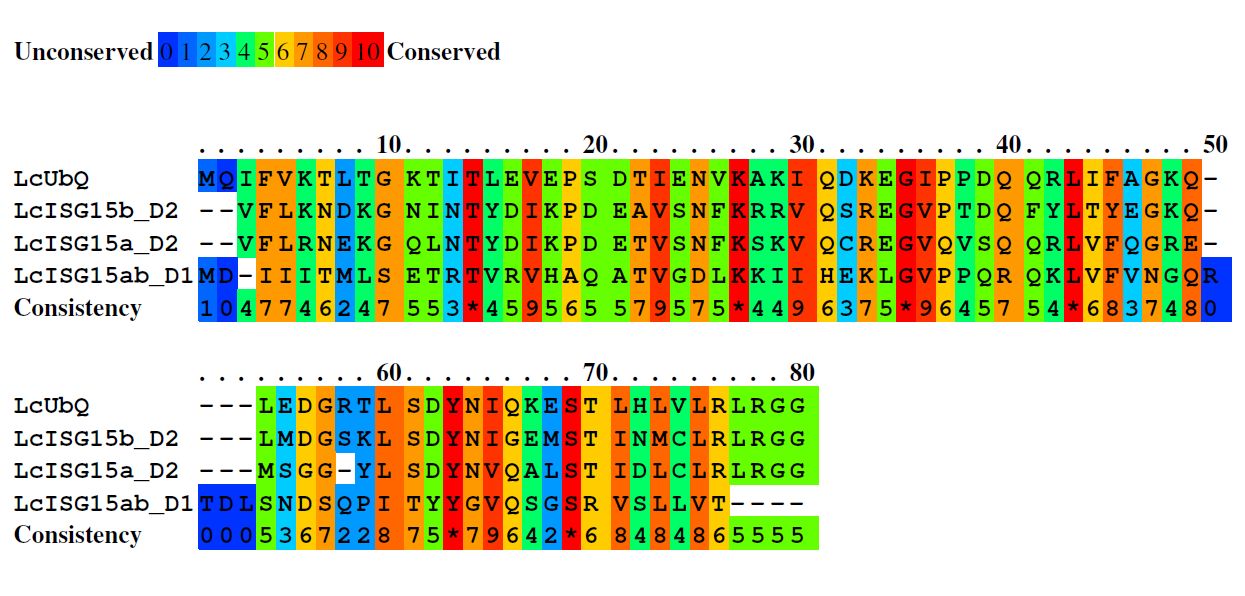


**B) Conservation of secondary structure**


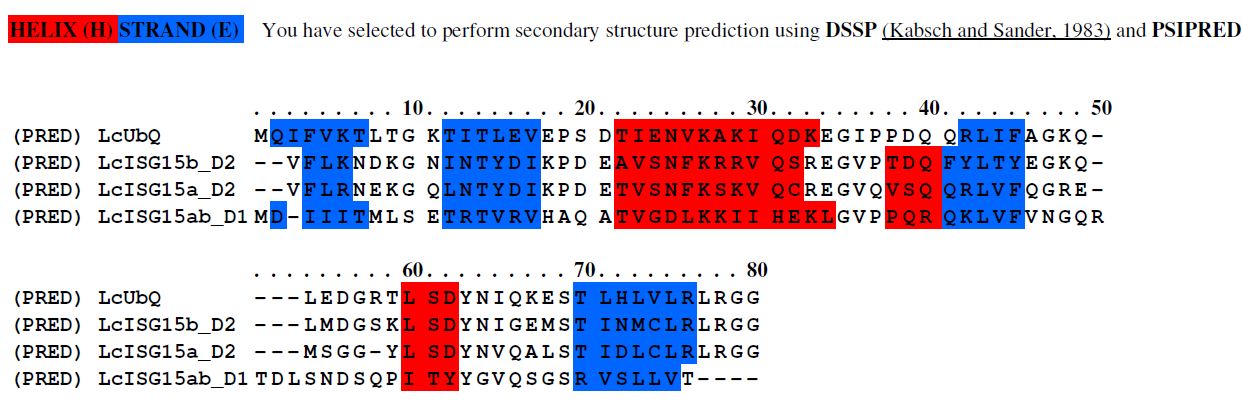


**Supplementary Figure 2:** **Similarity of *Lates* ISG15 with *Lates* Ubiquitin**. The Ubiquitin domains of *Lates* ISG15A and *Lates* ISG15B proteins were identified through SMART Domain prediction tool. The amino acids sequences domains of these ISG15 proteins were aligned and compared with the polyubiquitin-B sequence from *Lates* Accession No XP_018546613.1 (PREDICTED: polyubiquitin-B isoform X1 (*Lates calcarifer*). Alignments were done using PRALINE Server and residues coloured based on conservation (A) or secondary structure (B). As the first domain of LcISG15A and LcISG15B proteins were identical, only one sequence was used in this alignment (LcISG15ab D1).
